# Recombinant Infectious Bronchitis Viruses Expressing Chimeric Spike Glycoproteins Induce Partial Protective Immunity against Homologous Challenge despite Limited Replication *In Vivo*

**DOI:** 10.1128/JVI.01473-18

**Published:** 2018-11-12

**Authors:** Samantha Ellis, Sarah Keep, Paul Britton, Sjaak de Wit, Erica Bickerton, Lonneke Vervelde

**Affiliations:** a Infection and Immunity, The Roslin Institute, University of Edinburgh, Penicuik, Midlothian, United Kingdom; b The Pirbright Institute, Pirbright, Surrey, United Kingdom; c GD Animal Health, Deventer, The Netherlands; Loyola University Medical Center

**Keywords:** BeauR, S1, avian infectious bronchitis virus, coronavirus, partial protection, rIBV, recombinant vaccine, spike

## Abstract

Vaccination regimes against *Infectious bronchitis virus* (IBV), which are based on a single virus serotype, often induce insufficient levels of cross-protection against serotypes and two or more antigenically diverse vaccines are used in attempt to provide broader protection. Amino acid differences in the surface protein, spike (S), in particular the S1 subunit, are associated with poor cross-protection. Here, homologous vaccination trials with recombinant IBVs (rIBVs), based on the apathogenic strain, BeauR, were conducted to elucidate the role of S1 in protection. A single vaccination of specific-pathogen-free chickens with rIBV expressing S1 of virulent strains M41 or QX, BeauR-M41(S1) and BeauR-QX(S1), gave incomplete protection against homologous challenge, based on ciliary activity and clinical signs. There could be conformational issues with the spike if heterologous S1 and S2 are linked, suggesting a homologous S2 might be essential. To address this, a homologous vaccination-challenge trial incorporating rIBVs expressing full spike from M41, BeauR-M41(S), and S2 subunit from M41, BeauR-M41(S2) was conducted. All chimeric viruses grew to similar titers *in vitro*, induced virus-specific partial protective immunity, evident by cellular infiltrations, reductions in viral RNA load in the trachea and conjunctiva and higher serum anti-IBV titers. Collectively, these findings show that vaccination with rIBVs primed the birds for challenge but the viruses were cleared rapidly from the mucosal tissues in the head. Chimeric S1 and S2 viruses did not protect as effectively as BeauR-M41(S) based on ciliary activity and clinical signs. Booster vaccinations and an rIBV with improved *in vivo* replication may improve the levels of protection.

**IMPORTANCE** Infectious bronchitis virus causes an acute, highly contagious respiratory disease, responsible for significant economic losses to the poultry industry. Amino acid differences in the surface protein, spike (S), in particular the S1 subunit, have been associated with poor cross-protection. Available vaccines give poor cross-protection and rationally designed live attenuated vaccines, based on apathogenic BeauR, could address these. Here, to determine the role of S1 in protection, a series of homologous vaccination trials with rIBVs were conducted. Single vaccinations with chimeric rIBVs induced virus-specific partial protective immunity, characterized by reduction in viral load and serum antibody titers. However, BeauR-M41(S) was the only vaccination to improve the level of protection against clinical signs and the loss of tracheal ciliary activity. Growth characteristics show that all of the rIBVs replicated *in vitro* to similar levels. Booster vaccinations and an rIBV with improved *in vivo* replication may improve the levels of protection.

## Abstract (precis)

Infectious bronchitis virus causes an acute, highly contagious respiratory disease, responsible for significant economic losses to the poultry industry. Amino acid differences in the surface protein, spike (S), in particular the S1 subunit, have been associated with poor cross-protection. Available vaccines give poor cross-protection and rationally designed live attenuated vaccines, based on apathogenic BeauR, could address these. Here, to determine the role of S1 in protection, a series of homologous vaccination trials with rIBVs were conducted. Single vaccinations with chimeric rIBVs induced virus-specific partial protective immunity, characterized by reduction in viral load and serum antibody titers. However, BeauR-M41(S) was the only vaccination to improve the level of protection against clinical signs and the loss of tracheal ciliary activity. Growth characteristics show that all of the rIBVs replicated *in vitro* to similar levels. Booster vaccinations and an rIBV with improved *in vivo* replication may improve the levels of protection.

## INTRODUCTION

Infectious bronchitis virus (IBV) is classified as a *Gammacoronavirus*, subfamily *Coronavirinae*, order *Nidovirales* (1). IBV is responsible for major economic losses to poultry industries worldwide as a result of poor weight gain, decreased egg production, and impaired egg quality. The effect of IBV on the ciliary activity in the trachea and the immune system may predispose infected chickens to secondary infections with opportunistic bacteria, which often increases the mortality rate associated with IBV (2–4).

IBV is an enveloped virus, with a single-stranded, positive-sense RNA genome (∼28 kb) and encodes four structural proteins: nucleocapsid protein (N), spike glycoprotein (S), small membrane protein envelope (E), and integral membrane protein (M) (5, 6). The major surface protein of IBV, S, is a type 1 glycoprotein which oligomerizes to form trimers (7) and is thought to be the main inducer of protective immunity (8–12). The S protein is proteolytically cleaved into two subunits, the N-terminal subunit S1 (approximately 500 to 550 amino acids, 90 kDa) and the C-terminal subunit, S2 (630 amino acids, 84 kDa), which contains the transmembrane domain. The S1 subunit plays a critical role in binding to cellular receptors since it contains the receptor binding domain (13, 14), determines the virus serotype, and is responsible for the induction of neutralizing antibodies (14–16). Multiple studies have shown that recombinant S1 expressed in adenovirus and Newcastle disease virus vectors can induce a certain level of protection in specified-pathogen-free (SPF) chickens against challenge with wild-type virus (11, 17, 18).

Vaccine programs against IBV often include a combination of live or inactivated vaccines which are based on several dominant field serotypes of the virus. The current vaccines often induce insufficient cross-protection, and combinations of antigenically different vaccines are used in an effort to improve levels of protection (19). Alongside this, with the continual emergence of new field strains the control of IBV is persistently a significant problem to the poultry industry.

A reverse genetics system based on the avirulent strain of IBV Beaudette has been developed (20, 21). This system has many potential applications, such as to enhance our understanding of the role of individual genes in pathogenicity and to lead to a new generation of rationally designed live attenuated vaccines (20). Previous work using the reverse genetics approach demonstrated that replacement of the ectodomain of the S glycoprotein of the apathogenic IBV Beaudette strain with the same region from either of two pathogenic IBV strains, M41-CK or 4/91, resulted in two nonvirulent rIBVs, BeauR-M41(S) and BeauR-4/91(S), respectively. Notably, both rIBVs based on the BeauR backbone acquired the same cell tropism of that of the donor S, M41-CK or 4/91 (22, 23). Other work demonstrated that the Beaudette S2 subunit confers the unique ability of Beaudette to replicate in African green monkey kidney (Vero) cells, a continuous cell line licensed for vaccine production (24, 25). Vaccination with BeauR-M41(S) or BeauR-4/91(S) can confer protection against homologous challenge based on ciliary activity, reductions in clinical signs and viral load in the trachea at 5 days postchallenge (dpc), further demonstrating the dominant role of the S glycoprotein in inducing protective immunity (23, 26).

**FIG 1:**
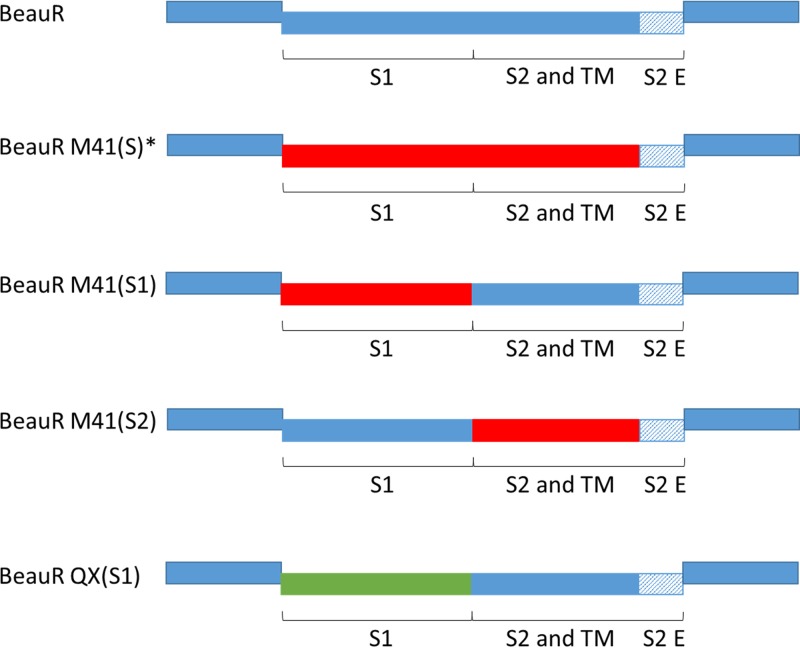
Design of rIBV constructs. Schematic of wild-type BeauR and rIBV genomes generated by reverse genetics to display homologous spike genes in Beaudette backbone. The rIBVs generated expressed either the S1 and/or S2 ectodomain and transmembrane domain (TM) from M41 and QX wild-type virus; with M41 derived genes represented by red boxes and QX derived genes represented by green boxes. In all rIBVs the Beaudette backbone is represented by solid blue boxes, and the endodomain (E) of S2 from Beaudette is represented by shaded blue boxes. *, BeauR-M41(S) displays the full ectodomain of M41 spike, as previously described (22).

In this study, we investigated the protection conferred against homologous challenge by two rIBVs, BeauR-M41(S1) and BeauR-QX(S1), that contain S1 subunits from economically relevant strains, M41 and QX, respectively, with the S2 subunit derived from BeauR (Fig. 1). Notably both rIBVs have the advantageous ability to replicate in Vero cells (26; E. Bickerton et al., unpublished data) due to the presence of the Beaudette S2 subunit. We report here on the first application of rIBV with a chimeric S gene to be used in a vaccination trial. The rIBV BeauR-M41(S2) was also investigated in order to elucidate the relevant roles of both subunits in protective immunity. While the S1 subunit is considered to be immunodominant, the S2 subunit is highly conserved between strains and contains immunogenic regions (14, 27).

We have shown here that vaccination with a recombinant IBV expressing a chimeric S gene can induce a partially protective response against challenge, as assessed by viral load, cellular infiltration, clinical signs, and a boost in serum antibody titers postchallenge. Vaccination with rIBV expressing homologous S1 and S2 subunits (i.e., full S gene) in the Beaudette backbone induced partial protection classified by the level of ciliary activity and presence of clinical signs following challenge with wild-type IBV. Comparison of *in vitro* growth characteristics shows that inclusion of a foreign S gene or a chimeric S gene in the rIBVs does not impede replication *in vitro*. However, our data show that despite the ability to induce a degree of virus-specific protective immunity, the rIBVs are hindered by limited *in vivo* replication and the attenuated BeauR backbone.

## RESULTS

### Characterization of rIBV BeauR-M41(S1) and BeauR-QX(S1) for homologous protection.

To determine whether a single vaccination with rIBV expressing the S1 subunit of the S gene (with a Beaudette derived S2 subunit) was sufficient to induce protection against challenge with homologous pathogenic isolates of IBV, a vaccination/challenge trial was conducted with BeauR-M41(S1) and BeauR-QX(S1). No clinical signs nor loss of ciliary activity in the trachea were observed in either of the vaccinated groups after vaccination (data not shown). These results showed that replacement of the BeauR S1 gene with the S1 gene from pathogenic strains did not confer pathogenicity to the resulting BeauR-M41(S1) and BeauR-QX(S1) viruses.

**FIG 2:**
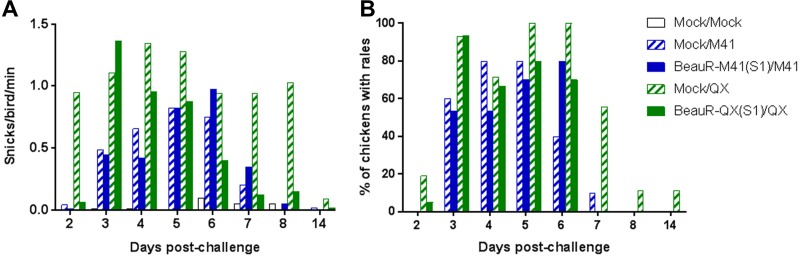
Assessment of clinical signs associated with BeauR-M41(S1) and BeauR-QX(S1) vaccination after challenge with M41-CK or QX. The findings for snicking (A) and rales (B) (*n* = 10 to 20 per group) are depicted.

Three weeks after the primary inoculation, chickens were challenged with a homologous wild-type virus strain, M41-CK or QX. Clinical signs were at the highest level in the challenge control groups, with QX more pathogenic than M41-CK (Fig. 2A and B). The rIBV vaccines expressing the S1 subunit did not confer full protection against clinical signs associated with IBV, although snicking and rales in the group vaccinated with QX(S1) resolved quicker than the QX challenge control (Fig. 2A and B). Vaccination with BeauR-M41(S1) or BeauR-QX(S1) did not prevent the loss of ciliary activity in the trachea following challenge with the homologous wild-type virus (Table 1).

**TABLE 1:** Assessment of protection against ciliostasis associated with BeauR-M41(S1) and BeauR-QX(S1) vaccination following challenge with M41-CK or QX

Vaccination/challenge	% ciliary activity (mean ± SD)^*a*^	No. of birds with 90% ciliary activity/total birds examined^*b*^	% of group protected^*c*^
Mock/Mock	92 ± 8.2	5/5	NA
Mock/M41	2 ± 1.4	0/5	0
BeauR-M41(S1)/M41	9 ± 16.3	0/5	0
Mock/QX	1 ± 0	0/5	0
BeauR-QX(S1)/QX	1 ± 1.4	0/5	0

a That is, the mean ciliary activity per group calculated from ciliostasis scores for 10 tracheal rings per individual bird using the following formula: [(total ciliostasis score of tracheal rings)/40] × 100.

b Ciliary activity was assessed according to European Pharmacopeia standards (27), wherein a bird is deemed protected against ciliostasis if no fewer than 9 of 10 tracheal rings per bird show normal ciliary activity (>50% ciliary activity retained).

c A vaccine is considered efficacious at conferring protection against ciliostasis when 80% or more of the birds in a group were protected. NA, not applicable.

**FIG 3:**
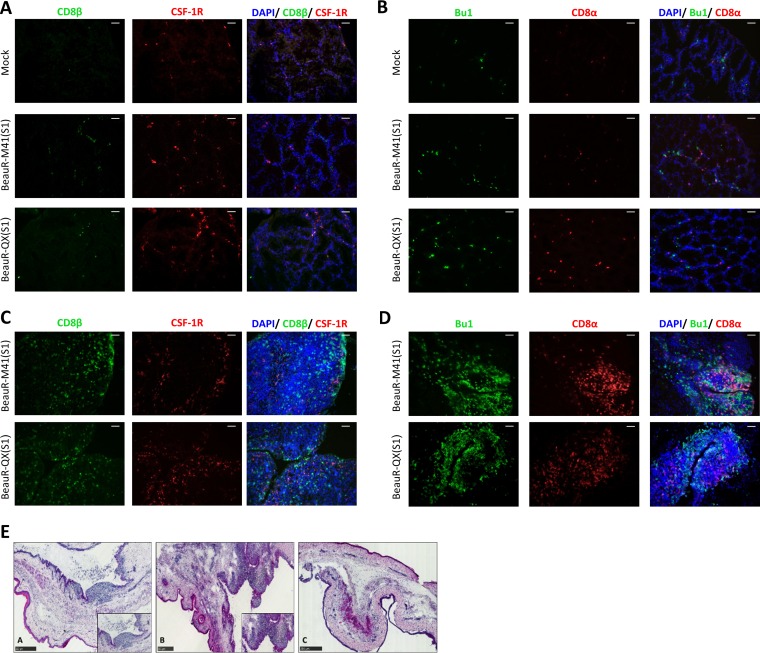
Cellular infiltrates of head-associated lymphoid tissues following vaccination with BeauR-M41(S1) and BeauR-QX(S1). (A and B) Harderian glands at 2 dpv. (C to E) CALT tissues at 2 dpv. Cryosections were stained with monoclonal antibodies to detect CSF-1R^+^ (red) and CD8β^+^ (green) cells (A and C) or to detect Bu-1^+^ (green) and CD8α^+^ (red) cells (B and D). Nuclei were labeled with DAPI (blue). Scale bars, 50 µm. (E) H&E-stained cryosections of the lower conjunctiva. Inset images depict the CALT regions detected in BeauR-M41(S) (see panel A) or BeauR-QX(S1) (see panel B) tissues which were not clearly evident in mock-treated lower conjunctiva (C). Scale bars, 250 µm. Representative images are shown in all panels.

To investigate the tissue tropism of the rIBVs, a range of tissues collected at 2 and 4 days postvaccination (dpv) were assessed by reverse transcription-PCR (RT-PCR). BeauR-M41(S1) and BeauR-QX(S1) RNA was not detected in the conjunctiva, Harderian gland, nasal mucosa-associated lymphoid tissue (NALT) or trachea at 2 and 4 dpv (data not shown). Histological analysis of the head-associated lymphoid tissues revealed cellular infiltrates in both the Harderian gland and the conjunctiva-associated lymphoid tissue (CALT) at 2 dpv (Fig. 3A to D), with areas of CALT more prominent in vaccinated tissues compared to mock-treated samples (Mock) (Fig. 3E). Collectively, these findings suggest that the recombinant vaccine viruses did infect these tissues but were no longer detectable by PCR at 2 dpv, suggesting rapid clearance from the sites of inoculation and mucosal tissues in the head-associated lymphoid tissues exerted by a virus-specific protective immune response.

**FIG 4:**
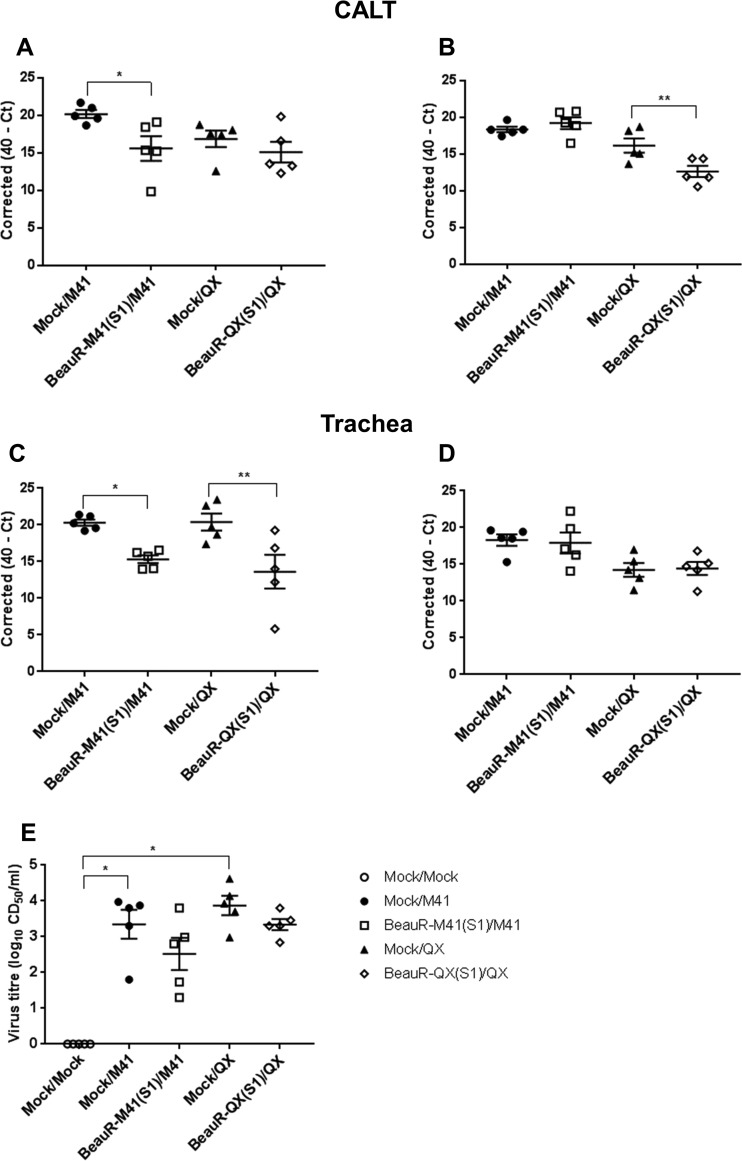
Viral load in the CALT and trachea in BeauR-M41(S1)- and BeauR-QX(S1)-vaccinated chickens after challenge with M41-CK or QX. (A to D) Relative viral RNA loads (expressed as corrected 40-*C_T_* values) at specific time points: 2 dpc (A and C) and 4 dpc (B and D). (E) Infectious viral load titers in the trachea at 4 dpc. Data points are shown as individual animals, and lines represent means and standard errors of mean (SEM). Statistically significant differences between groups are highlighted. *, *P* <0.05; **, *P *<* *0.01.

To elucidate whether BeauR-M41(S1) and BeauR-QX(S1) were able to confer a degree of protection against homologous challenge, as evidenced by a reduction in viral load in infected tissues postchallenge, qPCR was conducted to assess the level of viral RNA in the trachea and CALT. At 2 dpc, the IBV viral RNA loads in both the trachea and the CALT were significantly lower in the BeauR-M41(S1)-vaccinated groups than in the challenge controls (Fig. 4A and C), but at 4 dpc the viral RNA load was only significantly lower in the CALT of the BeauR-QX(S1)-vaccinated group (Fig. 4B and D). The infectious viral load determined by titration of trachea tissue supernatant in tracheal organ cultures (TOCs) showed a reduction in infectious virions recovered from BeauR-M41(S1)- and BeauR-QX(S1)-vaccinated chickens, although this reduction was not significant compared to corresponding wild-type controls [BeauR-M41(S1), *P *=* *0.961; BeauR-QX(S1), *P *=* *0.999] (Fig. 4E). The wild-type control groups were the only groups to report significantly higher infectious viral loads recovered from the trachea compared to those of the Mock/Mock controls (Fig. 4E).

**FIG 5:**
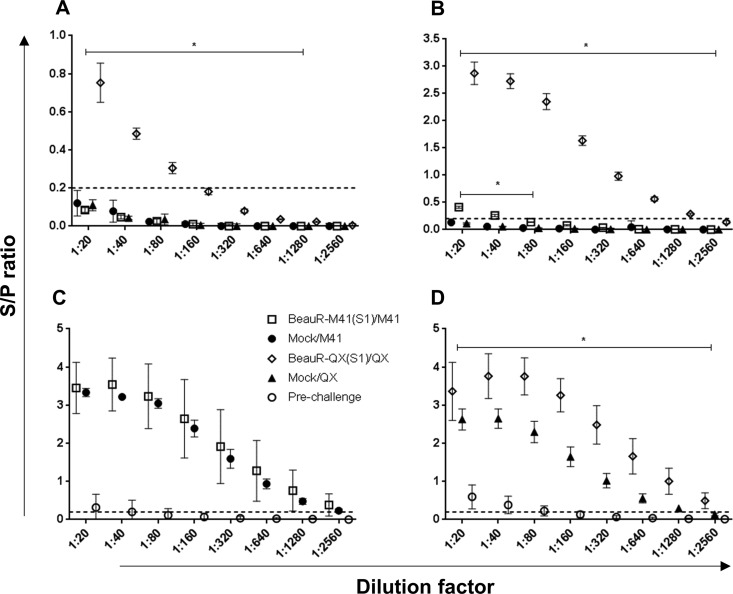
Measurement of serum anti-IBV titers of BeauR-M41(S1)- and BeauR-QX(S1)-vaccinated groups. Serum titers were assessed by commercial ELISA at 2 dpc (A) and 4 dpc (B) and in M41 groups at 14 dpc (C) and QX groups at 14 dpc (D). Prechallenge titers (i.e., 21 dpv) are included in panels C and D. The mean S/P ± the standard deviation from each group (*n* = 5 to 10) and includes four technical replicates/animal. Dashed line shows the cutoff for positive samples (S/P = 0.2). Solid bars denote a trend in statistical significance across dilutions in comparisons with the Mock/challenge-only group, e.g., BeauR-QX(S1)/QX compared to Mock/QX and BeauR-M41(S1)/M41 compared to Mock/M41. *, *P *<* *0.05.

Serum IBV-specific antibodies were assessed postvaccination (prechallenge) at 21 dpv and at 2, 4, and 14 dpc. Compared to the challenge control group, titers were significantly higher in the BeauR-QX(S1)-vaccinated group at 2 and 4 dpc (Fig. 5A and B) (*P *<* *0.05 and *P *<* *0.01, respectively). At 14 dpc, serum titers were higher in both the BeauR-M41(S1)- and BeauR-QX(S1)-vaccinated groups compared to the challenge control groups, but only the QX-vaccinated group was significantly higher compared to the corresponding challenge control group (Fig. 5C and D) (*P *<* *0.05). For both vaccinated groups, antibody titers at 21 dpv (prechallenge) could be classed as “borderline” positive due to being above the limits of the S/P cutoff (Fig. 5C and D).

In summary, results from trial 1 suggest that although vaccination of the chickens with BeauR-M41(S1) and BeauR-QX(S1) did not confer complete protection against homologous challenge based on clinical signs and ciliary activity, a single vaccination of young chickens induced a partially protective virus-specific immune response, as indicated by a significant reduction in the viral load in the trachea and the CALT. Higher IBV-specific serum antibody titers compared to challenge-only controls shows that vaccination with chimeric rIBVs was able to prime the birds for challenge. Whether the lack of full protection against the loss of ciliary activity and clinical signs was due to the absence of a homologous S2 subunit or an incorrect folding of M41/QX (S1) and BeauR (S2)—and therefore lower infectivity—could not be answered in this study. Therefore, a second trial addressing the issue of whether a homologous S2 is required for protection was conducted.

### Relative contribution of S1 and S2 to homologous protection.

**FIG 6:**
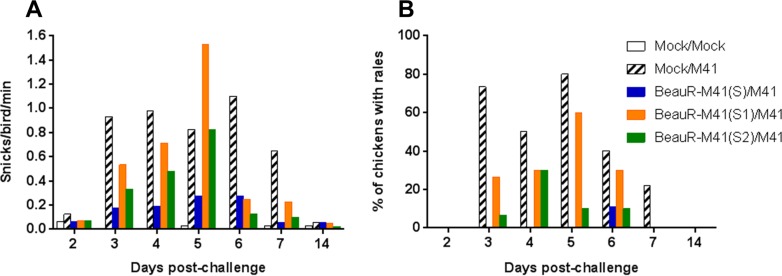
Assessment of clinical signs associated with BeauR-M41(S), BeauR-M41(S1), and BeauR-M41(S2) vaccination after challenge with M41-CK. The results for snicking (A) and rales (B) (*n* = 10 to 20 per group) are shown.

In trial 2, the rIBVs used were BeauR-M41(S), BeauR-M41(S1), and BeauR-M41(S2) (described in Fig. 1), with an experimental design similar to that used for trial 1. No clinical signs were observed in any of the vaccinated groups after vaccination (data not shown). After vaccination, there was no loss of ciliary activity in the trachea, indicating the apathogenicity of the rIBVs (data not shown). Similar to trial 1, at 21 dpv the chickens were challenged with M41-CK. Clinical signs were observed until 7 dpc; BeauR-M41(S) was the only vaccinated group to show less prevalent clinical signs postchallenge compared to the M41-CK challenge control (Fig. 6A and B). There was little difference between the BeauR-M41(S1), BeauR-M41(S2), and M41-CK groups in terms of the presence and severity of clinical signs (Fig. 6A and B), but in the vaccinated groups clinical signs resolved more rapidly compared to the M41-CK controls. Ciliary activity was assessed at 4 dpc, and the level of protection afforded were assessed according to European Pharmacopeia standards (28). The BeauR-M41(S)-vaccinated group retained ∼60% ciliary activity, showing an improved level of protection in comparison to groups vaccinated with BeauR-M41(S1) and BeauR-M41(S2), in which 20% protection in each group were evident (Table 2). Noteworthy, assessment on an individual bird level showed that three of five birds in the BeauR-M41(S) were classed as “protected against ciliostasis”; however, since the group average was 60%, this does not translate into protection on a group level (Table 2).

**TABLE 2:** Assessment of protection against ciliostasis associated with BeauR-M41(S), BeauR-M41(S1), and BeauR-M41(S2) vaccination following challenge with M41-CK

Vaccination/challenge	% ciliary activity (mean ± SD)^*a*^	No. of birds with 90% ciliary activity/total no. of birds examined^*b*^	% of group protected^*c*^
Mock/Mock	96 ± 5.2	5/5	NA
Mock/M41	0 ± 0	0/5	0
BeauR-M41(S)/M41	65 ± 36.2	3/5	60
BeauR-M41(S1)/M41	19 ± 33	1/5	20
BeauR-M41(S2)/M41	23 ± 43.4	1/5	20

a That is, the mean ciliary activity per group calculated from ciliostasis scores for 10 tracheal rings per individual bird using the following formula: [(total ciliostasis score of tracheal rings)/40] × 100.

b Ciliary activity assessed according to European Pharmacopeia standards (27), wherein bird is deemed protected against ciliostasis if no fewer than 9 of 10 tracheal rings per bird showed normal ciliary activity (>50% ciliary activity retained).

c The vaccine is considered to be efficacious at conferring protection against ciliostasis when 80% or more of the birds in a group were protected. NA, not applicable.

**FIG 7:**
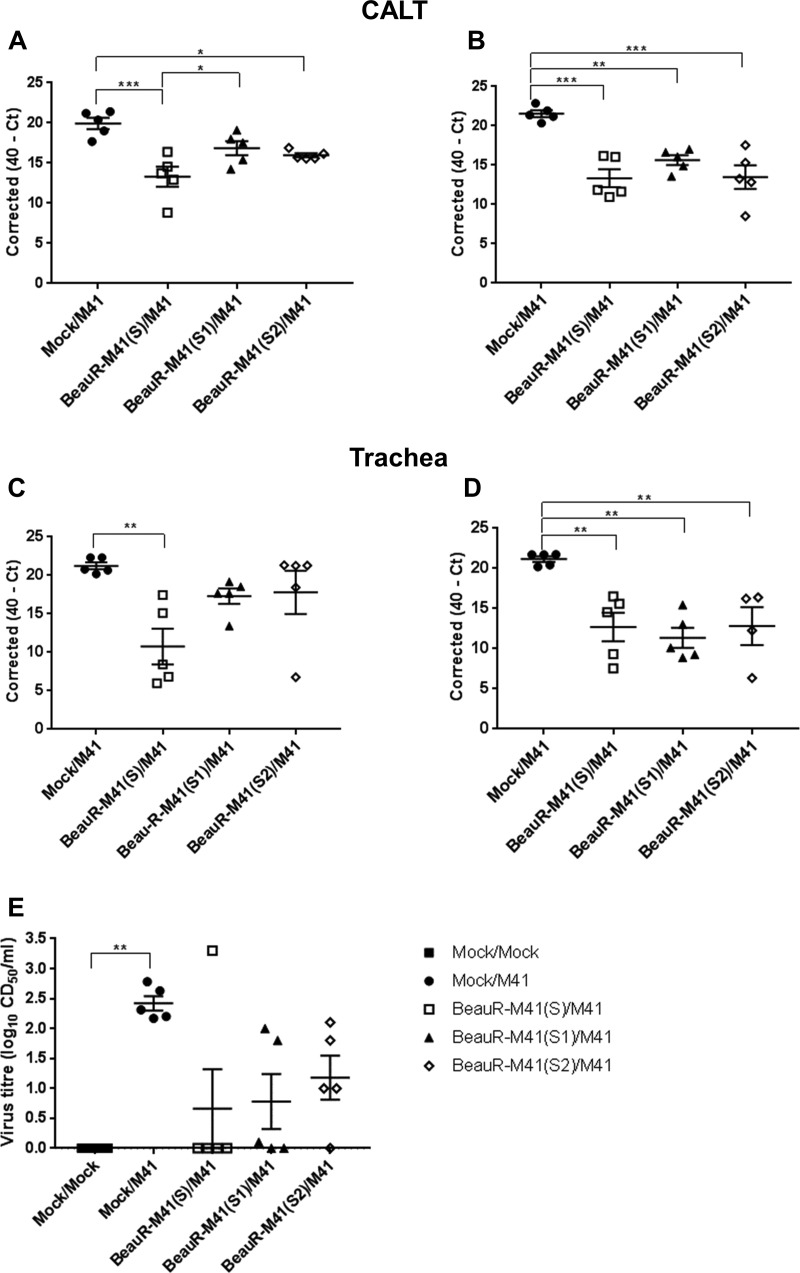
Viral load in the CALT and trachea in BeauR-M41(S)-, BeauR-M41(S1)-, and BeauR-M41(S2)-vaccinated chickens following challenge with M41-CK. (A to D) Relative viral RNA loads (expressed as corrected 40-*C_T_*) at 2 dpc (A and C) and 4 dpc (B and D). (E) Infectious viral load titers in trachea (4 dpc). Data points are shown as individual animals, and lines represent means ± SEM. Statistically significant differences between groups are highlighted. *, *P *<* *0.05; **, *P *<* *0.01; ***, *P *<* *0.001.

Viral RNA loads in the tracheas and CALTs isolated from challenged chickens were determined by qPCR to elucidate whether the S1 and S2 subunits played any further role in conferring protection. At 2 dpc only the CALT from BeauR-M41(S)- and BeauR-M41(S2)-vaccinated chickens showed any significant reduction (*P *<* *0.001) in viral RNA load compared to the challenge control (Fig. 7A). However, at 4 dpc all groups had significantly lower viral RNA loads in the CALT (Fig. 7B) [*P *<* *0.001, BeauR-M41(S); *P *<* *0.01, BeauR-M41(S1) and BeauR-M41(S2)]. Viral RNA loads in the trachea were only significantly lower at 2 dpc in BeauR-M41(S)-vaccinated chickens [*P *<* *0.001, BeauR-M41(S)] and significantly lower for all vaccinated groups at 4 dpc (Fig. 7C and D) [*P *<* *0.05, BeauR-M41(S) and BeauR-M41(S2), *P *<* *0.01, BeauR-M41(S1)]. Failure to locate the rIBVs in the head-associated lymphoid and respiratory tissues at 2 dpv in trial 1 led to the inclusion of the 1-dpv time point in trial 2. BeauR-M41(S), BeauR-M41(S1), and BeauR-M41(S2) were detected by RT-PCR in a number of the Harderian glands and tracheas isolated from chickens at 1 dpv; however, at 2 and 4 dpv the rIBVs were mainly detected in the nasal turbinates (Table 3), suggesting rapid clearance of rIBVs from the mucosal head tissues and sites of inoculation. Although the titers of infectious challenge virus recovered from tracheas at 4 dpc were not significantly reduced in BeauR-M41(S)-, BeauR-M41(S1)-, and BeauR-M41(S2)-vaccinated chickens compared to controls (because of the variation within each group), there was a general trend that vaccination resulted in a reduction in viral infectivity, with no detected infectious virus recovered in four of five birds in the S group, three of five birds in the S1 group, and one of five birds in the S2 group (Fig. 7E). Collectively, this shows that the chimeric rIBVs are able to induce a degree of local protection against the replication of IBV in the trachea.

**TABLE 3:** Detection of IBV-derived RNA by RT-PCR in head-associated lymphoid tissues and tracheal samples following vaccination with BeauR-M41(S), BeauR-M41(S1), and BeauR-M41(S2)^*a*^

Vaccination	dpv	No. of virus-positive tissues per group		
		Harderian gland	Trachea	Nasal turbinates
Mock	1	0/5	0/5	NA
BeauR-M41(S)	1	3/5	0/5	NA
BeauR-M41(S1)	1	1/5	0/5	NA
BeauR-M41(S2)	1	1/5	2/5	NA
Mock	2	0/5	0/5	0/5
BeauR-M41(S)	2	1/5	0/5	3/5
BeauR-M41(S1)	2	0/5	0/5	3/5
BeauR-M41(S2)	2	0/5	0/5	2/5
Mock	4	0/5	0/5	0/5
BeauR-M41(S)	4	3/5	0/5	2/5
BeauR-M41(S1)	4	1/5	0/5	2/5
BeauR-M41(S2)	4	1/5	2/5	3/5

a The results are depicted as the number of positive samples/number of birds per group (total of five birds/group). All positive results were confirmed by sequencing of PCR products (data not shown). NA, not applicable.

**FIG 8:**
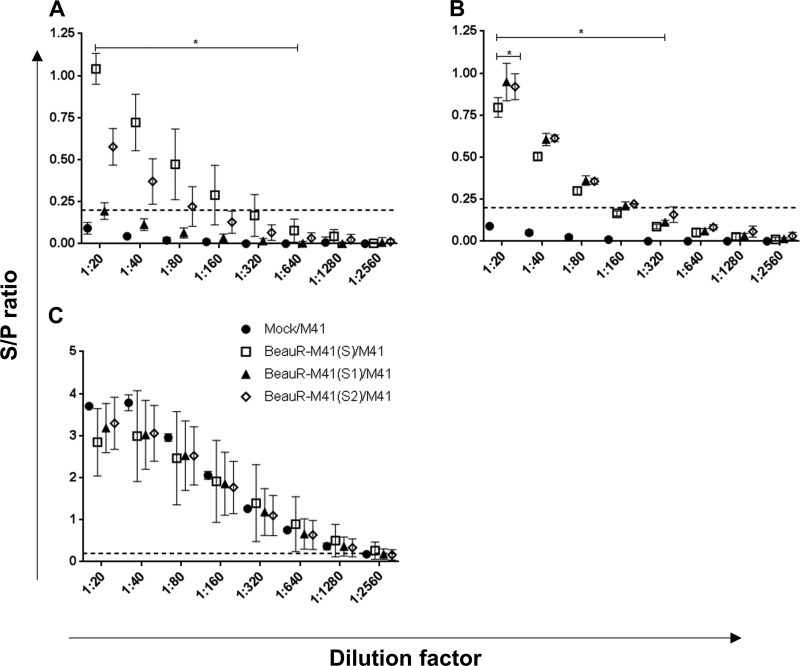
Measurement of serum anti-IBV titers of BeauR-M41(S)-, BeauR-M41(S1)-, and BeauR-M41(S2)-vaccinated groups. Serum titers were assessed by commercial ELISA at 2 dpc (A), 4 dpc (B), and 14 dpc (C). The mean S/P ratio ± SEM from each group (*n* = 10) includes four technical replicates/animal. Dashed line shows the cutoff for positive samples (S/P = 0.2). Solid bars denote a trend in statistical significance across dilutions in comparisons with the Mock/challenge-only group, e.g., BeauR-M41(S), BeauR-M41(S1), and BeauR-M41(S2) compared to Mock/M41. *, *P *<* *0.05.

To assess whether the rIBVs induced humoral antibody responses following vaccination with BeauR-M41(S), BeauR-M41(S1), and BeauR-M41(S2) viruses, IBV-specific serum titers were assessed at 2 and 4 dpc. At 2 dpc, there was clear evidence of a boost in antibody titers in the BeauR-M41(S)- and BeauR-M41(S2)-vaccinated groups (Fig. 8A), with significantly higher titers compared to Mock/M41 controls (*P *<* *0.001). IBV induced antibody titers at 2 dpc in BeauR-M41(S)-vaccinated chickens were higher than those from BeauR-M41(S1)- and BeauR-M41(S2)-vaccinated chickens across the dilution series (Fig. 8A). At 4 dpc, serum antibody titers from all vaccinated groups were significantly higher compared to the Mock/M41 titers (Fig. 8B), an observation suggestive of a primed antibody response in the vaccinated chickens. The serum antibody titers at 14 dpc indicated no significant differences between the vaccinated groups and the challenge-only controls (Fig. 8C), suggesting that a boosted response was lacking in response to challenge with wild-type virus.

**FIG 9:**
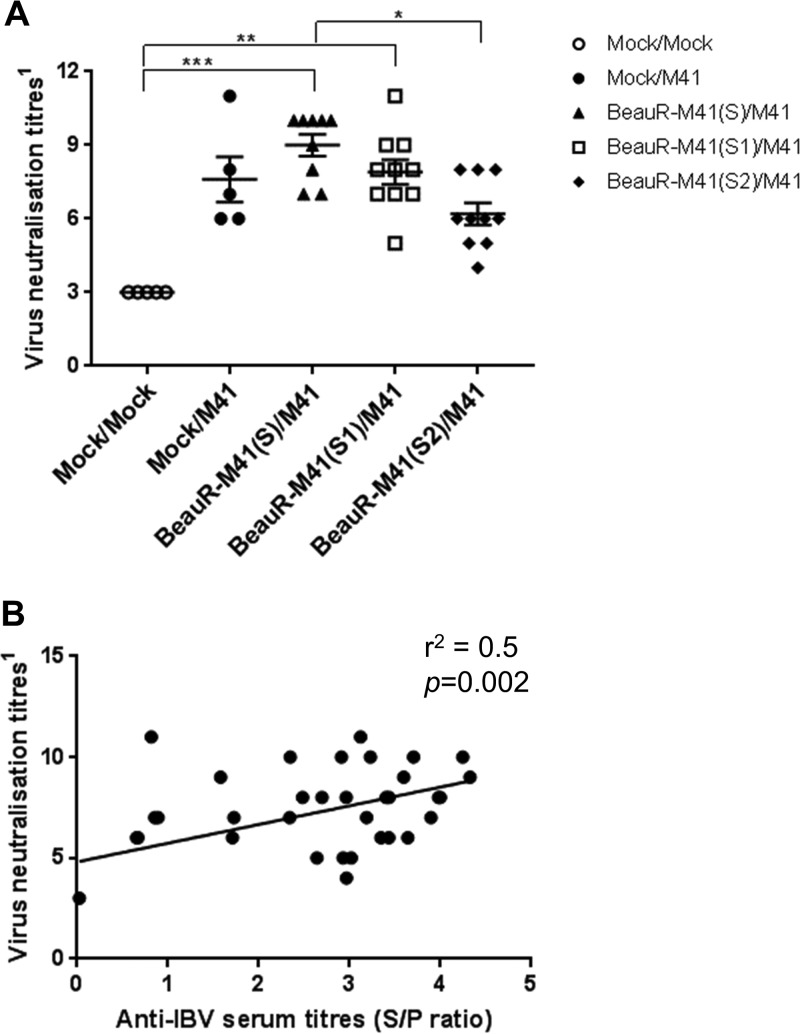
Measurement of virus neutralization antibody titers of BeauR-M41(S)-, BeauR-M41(S1)-, and BeauR-M41(S2)-vaccinated and Mock groups at 14 dpc. (A) Virus neutralization titers were determined by titration of serum in CK cells. Virus neutralization titers expressed as the log_2_ of the reciprocal of the highest serum dilution that showed complete inhibition of CPE (*n* = 5 or 10). Lines represent means ± SEM. Statistically significant differences between groups are highlighted. *, *P *<* *0.05; **, *P *<* *0.01; ***, *P *<* *0.001. (B) Relationship between virus neutralization activity and anti-IBV serum titers at 14 dpc. Data points represent the S/P ratios from individual serum samples (*n* = 39) plotted against virus neutralization titers, compared by Spearman rank correlation analysis.

The virus neutralization activity of the serum collected at 4 and 14 dpc were assessed, and at 4 dpc there was no neutralization of the virus detected (data not shown). At 14 dpc, only serum from BeauR-M41(S)- and BeauR-M41(S1)-vaccinated chickens had significantly higher neutralization activity of the virus compared to Mock/Mock controls (*P *=* *0.002 and *P *=* *0.0066, respectively; Fig. 9A). BeauR-M41(S) vaccination induced significantly higher virus neutralization titers compared to BeauR-M41(S2) vaccination (*P *=* *0.04), whereas there was no significant difference in titers compared with serum from BeauR-M41(S1)-vaccinated chickens or the Mock/M41 challenge-only group (Fig. 9A). The levels of virus neutralization activity detected were moderately positively correlated to the anti-IBV serum titers (*r*^2^= 0.5, *P *=* *0.002, Fig. 9B).

### Characterisation of rIBVs *in vitro*.

**FIG 10:**
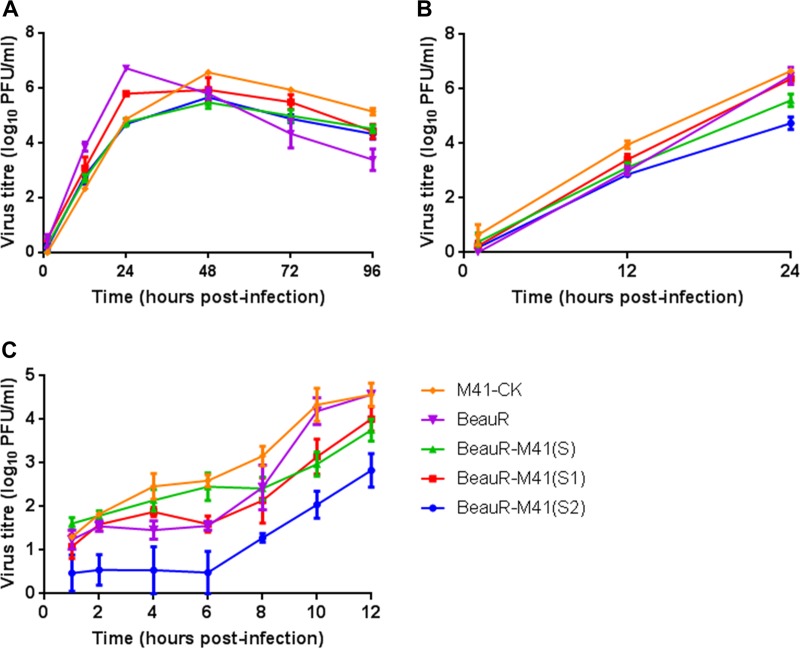
Comparison of the growth curves of BeauR-M41(S), BeauR-M41(S1), and BeauR-M41(S2). Multistep growth curves in CK cells derived from Valo chickens (A), 24-h growth curves (B), and single-step, 12-h growth curves (C) in CK cells derived from RIR chickens are depicted. Supernatant was harvested at various time points postinfection, and titers of progeny virus were determined by a plaque titration assay on CK cells. Data points represent the means of three independent experiments, and error bars represent the SEM.

Following on from the observation of differences during the *in vivo* vaccination trials, to elucidate whether the inclusion of a chimeric S gene or a foreign S gene had an effect on viral replication, the replication kinetics of rIBV BeauR-M41(S), BeauR-M41(S1), and BeauR-M41(S2) viruses were investigated *in vitro*. At 12 h postinfection (hpi) all viruses had similar titers (Fig. 10A and B). This suggests that the inclusion of a foreign S gene or a chimeric S gene has not impeded replication *in vitro* in either chicken kidney cells (CKCs) derived from Valo chickens (Fig. 10A) or CKCs derived from Rhode Island Red (RIR) birds (Fig. 10B). Single-step growth curves performed in CKCs derived from RIR birds show that over the latent period (2 to 8 hpi), there were lower virus titers for BeauR-M41(S2) compared to the other viruses; however, when exponential growth was compared, there was no statistical difference between the viruses (Fig. 10C). The titers of BeauR-M41(S) and BeauR-M41(S1) were similar across all time points (Fig. 10C).

## DISCUSSION

We have previously shown that rIBVs expressing the ectodomain of the Spike protein of a pathogenic strain in the context of an apathogenic strain BeauR could induce increased levels of protection against homologous and partially against heterologous challenge infection. Here, we extended this work and replaced only the S1 subunit of the ectodomain of BeauR with the S1 domain of M41 or QX, representing two strains that circulate in poultry flocks worldwide. These rIBVs have the advantage of being able to replicate in Vero cells, potentially allowing large-scale vaccine production in cell culture rather than in embryonated eggs. In this first vaccination study, using a single dose of BeauR-M41(S1) or BeauR-QX(S1) in 1-week-old chicks, the birds were not protected against homologous challenge based on ciliary activity and clinical signs. Vaccination with BeauR-QX(S1) induced significantly higher serum titers postchallenge, and the clinical signs associated with challenge virus, although present, decreased rapidly compared to unvaccinated birds challenged with QX. Together, these data show that vaccination with chimeric rIBVs are able to induce a degree of virus-specific immunity with partial local protection in the mucosal head tissues and the primary site of replication, the trachea.

To address the questions of whether a full homologous S is required for optimal folding, virus replication, and protection using an apathogenic recombinant virus, a second vaccination experiment was performed. One-week-old birds were immunized once with BeauR-M41(S), BeauR-M41(S1), or BeauR-M41(S2). Replacement of the apathogenic BeauR-S1 or S2 subunits with a S1 or S2 from a pathogenic strain, allowed BeauR to remain apathogenic, suggesting that the S1 or S2 alone do not play a role in the pathogenicity of IBV. This further expands our previous work showing that spike switching of BeauR-S with M41-S had no effect on pathogenicity (23). Here, vaccination of chickens with a rIBV based on a BeauR backbone expressing a full S gene from the donor serotype enhanced the level of protection afforded against tracheal ciliostasis, with three of five birds classed as fully protected. However, when classified under European Pharmacopeia standards for assessment of IBV vaccines (28), at which 80% protection (at a group level) against ciliostasis is required, the BeauR-M41(S)-vaccinated group was only able to confer partial protection (∼60%) and therefore is still not satisfactory for the criteria used for the assessment of IBV vaccines for industrial application. Consistent with previously published work, we show that collectively as a group the chickens vaccinated via ocular-nasal routes with BeauR-M41(S) had ∼60% ciliary activity remaining, a reduction in clinical signs, and viral load postchallenge (26). The protection seen at the trachea may potentially be improved with assessment of ciliostasis at a later time point, as Armesto et al. (23) reported that vaccination with BeauR-4/91(S) gave ∼60% ciliary activity at 4 dpc, which then improved to 90 to 100% at 6 dpc. In trial 1 the viral RNA load in the trachea and CALT from the S1-vaccinated groups was reduced at 2 dpc, whereas in trial 2 all vaccinated groups had a clear significant reduction in viral RNA loads at 2 and 4 dpc in the trachea and CALT. The qPCR used here is designed to detect the 5′ untranslated region (5′ UTR) of the genome (29), and it therefore may be detecting incomplete virions or challenge virus captured in the lumen of the trachea. To further support the viral RNA load data, infectious viral load recovered from the trachea in both trials 1 and 2 were lower in rIBV-vaccinated chickens, indicating a degree of local protection at the site of infection, which was not robust enough to completely protect against viral replication *in vivo* and the loss of ciliary activity.

The major surface glycoprotein of coronaviruses, spike, is a type 1 glycoprotein and has two structurally distinct conformations: prefusion and postfusion (30–32). In the coronavirus replication cycle, the spike mediates the critical steps of receptor binding and membrane fusion. Upon binding of the S1 receptor binding domain to the host cell, an irreversible conformational switch to the postfusion state allows the S2 subunit to fuse viral and cellular membranes, facilitating entry of the viral genome and therefore downstream viral replication (32–34). Recently, the crystal structure of the prefusion spike from mouse hepatitis virus and human coronavirus (HKU1) were resolved, highlighting the critical role that the interaction between the trimers of S1 and S2 plays in stabilization of the prefusion conformation of spike (31, 32). Here, expression of a chimeric spike in a recombinant IBV backbone with the lack of a homologous S2 possibly resulted in conformational changes either within the S1 subunit or complete S protein, potentially affecting receptor binding and entry, but may have also altered immunogenic epitopes. The S2 subunits of BeauR shares 87 and 97% amino acid sequence similarities with QX and M41-CK, respectively, showing that there are only a few different amino acid residues between them. The interactions between S1 and S2 subunits are critical for the maintenance of conformation, recognition, and efficient fusion of the spike to host cells; it has been consistently shown that even a single amino acid change within the S2 subunit of coronavirus spikes may influence the secondary structure of the overall spike or the S1 subunit (35, 36).

The development of a cryo-electron microscopic structure of IBV M41 spike, highlighting the evolutionary difference between the prefusion spike structures of IBV compared to betacoronaviruses and alphacoronaviruses, nonetheless indicates a high degree of structurally similarity to porcine deltacoronavirus (37, 38). This structural model of prefusion IBV spike will significantly aid in addressing the challenges over whether (i) expression of a chimeric spike in a rIBV backbone causes conformational changes either within the S1 subunit or complete S or (ii) it is vital that homologous “matched” S1 and S2 and their interactions are required to maintain the correct prefusion conformation of spike, as suggested in other coronaviruses.

The Beaudette strain, used here in the reverse genetics system, has an extended *in vitro* tropism, ability to grow in cell cultures and an apathogenic nature, making it an excellent resource for the investigation of heterologous genes and growth characteristics of rIBV. During embryo passages, however, the Beaudette strain may have acquired mutations which are likely to contribute to its lack of pathogenicity and restrict its *in vivo* tropism and replication. Replacement of the BeauR S1 or S2 with corresponding subunits from a pathogenic strain did not indicate a significant impairment of *in vitro* growth of the viruses in comparison to the BeauR virus, showing no indication that BeauR-M41(S1) and BeauR-M41(S2) were unable to enter the cells, fuse with cell membranes, or fail to replicate *in vitro*. Nevertheless, the lack of full protection afforded by BeauR rIBVs against wild-type challenge and the limited *in vivo* replication strongly suggest that attenuations have occurred in genes playing an essential role in replication and that these are negatively impacting its suitability as a vaccine vector. Development of an alternative, less-attenuated backbone for the expression of heterologous genes in rIBVs may promote the development of these live attenuated vaccines for the control of IBV.

Expression of the IBV S1 subunit alone has been shown to induce virus-neutralizing antibodies, albeit often requiring repeated vaccination (8, 10). Here, immunization of chickens with rIBVs based on the Beaudette backbone expressing either M41 S or chimeric S1/S2 induced virus-neutralizing antibodies; however, the Mock/M41 serum also had a degree of neutralizing activity. BeauR-M41(S) vaccinated chickens had significantly higher virus neutralizing titers than the BeauR-M41(S2) group, but there was no statistical difference with the BeauR-M41(S1) group, showing that neutralizing antibodies are induced following a single vaccination with rIBV expressing M41(S1).

Live attenuated vaccines against IBV need to induce a good level of mucosal immunity, with local tracheal and cell-mediated immunity also playing an important role in prevention of IBV infection (39–41). As discussed earlier, the BeauR backbone is impeded by poor *in vivo* replication, and the lack of protection shown against ciliostasis indicates that there is a poor level of local immunity induced in the trachea by vaccination with BeauR rIBVs. Cytotoxic responses can also play a key role in the early control of IBV, as indicated by previous studies showing NK cell activation (42), IBV-specific cytotoxic T-cell-lymphocyte (CTL) activity of splenocytes isolated from IBV-infected chickens (41), and higher CTL proportions in respiratory tissues following IBV infection (43). Cellular infiltrates in head-associated lymphoid tissues, as well as a reduction in viral load in the trachea and CALT, also imply that the rIBVs infected the chickens and suggests a possible role for the cell-mediated response. However, that we were unable to consistently detect the recombinant S1 viruses at 2 dpv raises possibilities that the viruses were either rapidly cleared from the tissues, replicate poorly at these sites of inoculation, or have limited replication in a few cells which are below detectable limits of the assays. In the BeauR-M41(S)-vaccinated group, more than 50% of the chickens were positive for vaccine virus, as assessed by RT-PCR, in the Harderian gland and nasal turbinates at 1 and 2 dpv, respectively. The primary site of IBV infection is thought to be the ciliated epithelium lining the trachea; however, following ocular-nasal vaccination, the virus has been detected in the nasal turbinates (44) and Harderian gland (45).

One possible explanation for the poor protection of ciliary activity afforded by the recombinant S1 viruses could be that we only administered a single vaccine dose to the SPF chicks. Previous studies using baculovirus expressed IBV recombinant proteins or IBV purified proteins have required multiple injections to achieve a degree of protection in SPF chickens (10, 17). There is also evidence of an impaired humoral response in young chicks with regard to IBV vaccination; vaccination of 1- and 7-day-old chicks showed a delay in both systemic and local IgA and IgG levels compared to vaccination of older chicks (14, 21, or 28 days old) (46). Here, in an attempt to improve the protection against respiratory signs and ciliostasis with the recombinant S1 viruses, a prime-boost approach may aid in overcoming these potential issues.

In summary, we have previously generated recombinant IBV based on a BeauR backbone expressing a heterologous S1 from M41 or QX, and in the present study we have shown that a single vaccination in young chicks with these rIBVs, although not adequate to completely prevent ciliostasis and clinical signs, can induce a degree of virus-specific protective immunity. This was characterized by a reduction in the viral load recovered from the trachea and CALT, cellular infiltrations at head mucosal and inoculation sites, higher serum antibody titers in vaccinated groups, and the induction of virus-neutralizing activity. Vaccination with BeauR-M41(S), despite expressing the homologous full S to attempt to overcome any issues with heterologous S1 and S2 subunits and suboptimal folding, only induce a partially protection against the loss of ciliary activity. Since the *in vitro* growth characteristics show that inclusion of a foreign S gene or a chimeric S gene in the rIBVs does not impede replication *in vitro*, perhaps the attenuated Beaudette backbone has hindered the *in vivo* replication of these rIBVs; thus, to improve protection, multiple vaccinations, or an alternative backbone may be required.

## MATERIALS AND METHODS

### Ethics statement.

All animal experimental protocols were carried out in strict accordance with the UK Home Office guidelines and under license granted for experiments involving regulated procedures on animals protected under the UK Animals (Scientific Procedures) Act 1986. The experiments were performed in The Pirbright Institute (TPI) Home Office licensed (X24684464) experimental animal house facilities and were approved by TPI’s animal welfare and ethical review committee under the terms of reference HO-ERP-01-1. Trial 1 used SPF RIR chickens obtained from TPI Poultry Production Unit in Compton. Trial 2 used the same chicken breed but obtained from The National Avian Research Facility in Edinburgh.

### Cells and viruses.

Tracheal organ cultures (TOCs) were prepared from 19-day-old SPF RIR chicken embryos (47–49). Primary chicken kidney (CK) cells were prepared by The Central Services Unit, TPI, from kidneys extracted from either 2- to 3-week-old SPF RIR chickens or 2-week-old SPF derived Valo chickens (49). The pathogenic M41 strain (50) used in this study had previously been adapted in CK cells to produce M41-CK (accession number X04722) (26). The pathogenic strain, QX (QX L1148 strain, accession number KY933090) (51), was donated by Richard Jones, University of Liverpool. The rIBVs BeauR-M41(S), BeauR-M41(S1), BeauR-M41(S2), and BeauR-QX(S1) used here are described in a schematic illustration (Fig. 1) and constructed using the backbone of Beau-R, which is the molecular clone of Beau-CK (accession number AJ311317) (21, 25). All isolates of IBV and rIBV were propagated in 10-day-old RIR SPF embryonated eggs. Allantoic fluid was clarified by low-speed centrifugation at 24 to 48 hpi. Titrations to determine virus infectivity were either performed in TOCs as described previously (26) or in CK cells (49); titers are expressed as 50% (median) ciliostatic doses (CD_50_)/ml or PFU/ml, respectively.

### Analysis of growth kinetics in CK cells.

Confluent CK cells seeded in either 6-well or 12-well plates were inoculated with 10^4^ PFU rIBV or IBV for multistep growth curves or 10^5^ PFU rIBV or IBV for single-step growth curves in 0.5 ml of serum-free *N*,*N*-bis(2-hydroxyethyl)-2aminoethanesulphonic acid (BES) medium and incubated for 1 h at 37°C at 5% CO_2_. Cells were washed with phosphate-buffered saline (PBS) to remove residual virus, and 2 ml of serum-free BES medium was added per well. Extracellular virus was harvested at defined intervals and assayed by titration in CK cells.

### Experimental design of *in vivo* vaccination/challenge trials.

SPF RIR chickens were housed in positive-pressure, HEPA-filtered isolation rooms in which each group was housed in a separate room. In two separate experiments, birds were randomly divided into 5 groups of 30 birds for trial 1 and 5 groups of 40 birds for trial 2. Eight-day-old chicks were inoculated (classified as primary inoculation) with 10^5^ PFU of BeauR-M41(S1) or BeauR-QX(S1) (trial 1) or 10^4^ PFU BeauR-M41(S), BeauR-M41(S1), or BeauR-M41(S2) (trial 2) in a total of 0.1 ml of PBS via conjunctival (eye drop) and intranasal routes. A challenge dose, equal to the primary inoculation, 10^5^ PFU (trial 1) and 10^4^ PFU (trial 2) of the corresponding wild-type viruses were administered in the same manner 21 days after the primary inoculation to the appropriate groups. Of note, the IBV QX strain used here could not be propagated in CK cells, so a CD_50_ dose of 10^2.73^ was used. Mock-infected controls were inoculated via the same route with 0.1 ml of PBS, and mock/challenge control groups were inoculated with 0.1 ml PBS and challenged with the same dose of wild-type virus. Birds were euthanized by cervical dislocation at specific times postinfection, and a panel of tissues was sampled to allow for downstream analysis. Blood samples were collected and processed for the collection of serum. The clinical signs used to determine pathogenicity were snicking, rales, and ciliary activity of the trachea (a bird was considered protected if 50% or more ciliary activity was retained in 9 of 10 tracheal rings; this must be in 80% of the group) (28, 52).

### Isolation of tissues: virus isolation and ciliostasis assay.

Tissues collected were divided into two parts; one part was stabilized in RNAlater (Ambion) for RNA extraction and the other in 20% sucrose–PBS (0.22-μm-pore size filtered) at 4°C overnight before snap freezing in OCT (Thermo Scientific) for histology. Tissues collected included: Harderian gland, CALT, NALT, and trachea. Tissues were removed at 2 and 4 days postvaccination (dpv) and at 2, 4, and 14 days postchallenge (dpc). Tracheas were removed from five randomly selected chickens from each group at 4 dpv and 4 dpc for assessment of ciliary activity as described previously (26). Part of the trachea and CALT tissues were stored in PBS for virus isolation.

### Detection of viral RNA.

For virus isolation and RNA extraction, tissues stored in PBS and RNAlater, respectively, were freeze-thawed and homogenized using a TissueLyser II (Qiagen), as described previously (23). Total RNA was isolated using an RNeasy Mini-Kit and DNase treated according to the manufacturer’s instructions (Qiagen). cDNA was synthesized from 1 µg of tRNA using Superscript IV reverse transcriptase (Life Technologies) with a random oligonucleotide primer according to the manufacturer’s instructions. To quantify infectious viral load in trachea, tissue-derived supernatant was titrated in TOCs. To determine whether infectious virus was present, 10-day-old SPF embryonated eggs were inoculated with 100 µl of allantoic fluid; then, at 24 to 48 hpi they were assessed for viral presence by RT-PCR using primers specific for the 3′ UTR, as described previously (53). For quantification of viral load, qPCR was performed using TaqMan Universal PCR Master Mix (Applied Biosystems) with primers and probes specific to the 5′-UTR region, as described previously (29). Serial dilutions of M41 cDNA (generated from 1 µg of tRNA) were included to generate a standard curve, and the data, expressed in terms of the cycle threshold (*C_T_*) value, were normalized using the *C_T_* value of the 28S cDNA product for the same sample (54).

### Infectious bronchitis virus ELISA.

Serum samples collected at 21 dpv (prechallenge) and at 2, 4, and 14 dpc were assayed with a commercial IDEXX IBV antibody test kit (IDEXX Laboratories). To determine the endpoint titer, the serum samples were twofold serially diluted in the range 1:20 to 1:2,560 prior to incubation. After sample incubation, the remaining steps were followed directly according to the manufacturer’s instructions. The sample/positive (S/P) ratio was calculated using by the following equation: [(mean sample – mean kit negative)/(mean kit positive – mean kit negative)]. S/P ratios greater than 0.2 were considered positive for IBV antibodies. Polyclonal chicken serum raised against M41 and QX serum were included on each independent test plate (GD Animal Health).

### Immunocytochemistry.

For fluorescent microscopy, cryostat sections (5 µm) were fixed in acetone, washed in PBS, and blocked for 1 h at room temperature with 10% normal goat serum and 0.5% bovine serum albumin in PBS (blocking buffer). Slides were washed and incubated for 1 h with optimally diluted primary antibodies (anti-Bu-1 [clone AV-20; AbD Serotec], anti-CD8α [clone 3-298, AbD Serotec], and anti-CD8β [clone EP42, AbD Serotec]) and anti-CSF1R (55) or isotype controls, all diluted in blocking buffer. Sections were washed, incubated with an Alexa Fluor 488-labeled goat anti-mouse IgG1/IgG2a or Alexa Fluor 568-labeled goat anti-mouse IgG1/IgG2b according to the appropriate isotype, and diluted in blocking buffer for 1 h. Nuclei were visualized using DAPI (4′,6′-diamidino-2-phenylindole (Invitrogen). Images were captured with a Leica DMLB fluorescence microscope with a coupled device digital camera and analyzed using ImageJ analysis software. For light microscopy, cryostat sections (5 µm) were fixed in acetone and stained with Harris’ hematoxylin (Sigma-Aldrich) and 1% eosin (Sigma-Aldrich). Sections were dehydrated through graded ethanols and xylene and mounted in a xylene-based medium (DePex; Gurr-BDH Chemicals). Images were captured with a Hamamatsu Nano-zoomer-XR digital slide scanner.

### Analysis of neutralizing antibody.

Virus neutralization tests were performed by GD Animal Health (56). Briefly, twofold serial dilutions of serum were made in a 1:1 mixture of Medium-199 and Ham F10 in 96-well plates. To each well, an equal volume of CEK cells (in medium supplemented with 10% fetal calf serum) was added. After culture with M41 for 3 to 4 days at 37°C with 5% CO_2_, cell monolayers were examined for cytopathic effect (CPE). All individual titers were expressed as the log_2_ value of the reciprocal of the highest serum dilution that showed complete inhibition of CPE.

### Statistical analyses.

Viral load qPCR data were tested for normality through residual plots, and the differences between the mean corrected 40-*C_T_* values were statistically evaluated by a parametric one-way analysis of variance test adjusted for *post hoc* analysis using Tukey’s pairwise comparison. Serum antibody levels, viral isolation titers, ciliary activity, and virus neutralization titers were tested for normality, and nonparametric analyses were performed. Differences between the groups were statistically evaluated by the nonparametric Kruskal-Wallis test adjusted for *post hoc* analysis and Mann-Whitney U pairwise comparison. The relationship between anti-IBV serum and virus neutralization titers were compared by Spearman rank correlation analysis. Analysis of the viral growth curves was conducted by fitting a polynomial curve to the exponential phase of viral growth (57); growth rates were then compared between groups by using the nonparametric Kruskal-Wallis test adjusted for *post hoc* analysis. For all statistical analyses, *P* values of <0.05 were considered significant. All statistical analyses were conducted using MiniTab v17 or GraphPad Prism 7.
